# Editorial: Precise Genome Editing Techniques and Applications

**DOI:** 10.3389/fgene.2020.00412

**Published:** 2020-04-21

**Authors:** Kun Xu, David Jay Segal, Zhiying Zhang

**Affiliations:** ^1^College of Animal Science and Technology, Northwest A&F University, Yangling, China; ^2^Department of Biochemistry and Molecular Medicine, Genome Center, University of California, Davis, Davis, CA, United States

**Keywords:** precise genome editing, CRISPR, HDR efficiency, biallelic HDR targeting, off-target effect, animal model, base editing

The CRISPR/Cas system, particularly CRISPR/Cas9 (Jinek et al., 2012; Cong et al., 2013), has been developed as a robust and versatile platform for manipulating the genomes of a variety of species. In recent years, numerous reports have suggested its powerful potential application for human gene therapy and life science research, as well as animal and plant breeding. This might be evidenced by the collections in this Research Topic, “Precise Genome Editing Techniques and Applications.”

Generally, the CRISPR/Cas9 nuclease is used to cleave target genomic DNA to generate site-specific double-strand breaks (DSBs), which are predominantly repaired via non-homologous end joining (NHEJ) or, to lesser extent, by homology-directed repair (HDR). The classical NHEJ repair pathway can generate small insertions or deletions (indels), resulting in loss-of-function of targeted coding genes by introducing a frameshift in the open reading frame (ORF). NHEJ mutagenesis is a highly popular strategy for gene manipulation. In addition to the classical NHEJ, alternative or accurate NHEJ-mediated repair can achieve precise genomic DNA deletions (Guo et al., 2018; Shou et al., 2018).

Two papers in this Research Topic by Chao et al. and Zhao et al. describe the manufacture of allele-specific knockout and double gene knockout mouse models for rapid disease gene validation and human xenograft studies, respectively. *N*^6^-methyladenosine (m^6^A) is a well-established epigenetic modification on eukaryotic mRNA. An increasing number of studies have uncovered the significance of m^6^A methylation, which has given rise the nascent field of “epitranscriptomics.” Another article in this volume (Huang et al.) describes a knock-out study in mouse spermatogonial GC-1 cells of the fat mass and obesity-associated (*Fto*) gene, which has been shown to act on the epitranscriptome as an m^6^A demethylase (Li et al., 2017; Lin et al., 2017).

On the other hand, the HDR repair pathway relies on homologous donor DNA to produce targeted gene knock-ins at the DSB site or gene replacement between two DSB sites. Precise point mutations and designed small indels can also be achieved by this method. One paper in this topic describes efforts to precisely correct the methyl-CpG binding protein 2 (MECP2) gene in the context of Rett syndrome (RTT) by CRISPR/Cas9-mediated HDR in human induced pluripotent stem cells (iPSCs). This report provides a reference for iPSC-based disease modeling and gene correction therapy (Le et al.).

Although the HDR-based genome can achieve gene insertions and precise substitutions, it is still confronted by several disadvantages during the precise editing process including low HDR efficiency, failure of biallelic targeting, complications of positive selection, and the re-deletion of selection markers.

It has been reported that inhibiting the key molecules of the competitive NHEJ pathway, such as DNA ligase IV (LIG4) and KU70, could improve the HDR efficiency effectively (Chu et al., 2015; Maruyama et al., 2015). We have previously developed a novel sgRNA-shRNA structure transcribing multiple sgRNAs for multiplex genome targeting (Yan et al., 2016). Here, the structure was further used for simultaneous *LIG4* RNA interference and the enhanced HDR-based *IGF2* SNP editing (Sun et al.). On the other hand, the HDR pathway can be also enhanced by the association of Cas9 with a variety of homologous recombination factors, such as yRad52 (Shao et al., 2017), dn53BP1 (Paulsen et al., 2017; Jayavaradhan et al., 2019), hRad51 (Rees et al., 2019), and CtIP (Tran et al.). A review paper in this topic (Liu et al.) further summarizes the methodologies and other considerations for improving the HDR efficiency.

Regarding biallelic targeting, we have previously reported a novel strategy using two donors with paired selectable markers (Wu et al., 2017). However, the removal of the selection is often required to allay concerns of marker-dependent effects. There are several “pop in and out” two-step techniques for marker-free genome engineering, including the Cre/LoxP system (Zhu et al., 2015), the *piggyBac* transposon (Xie et al., 2014), and the SSA repair mechanism (Li et al., 2018). This Research Topic presents a protocol article for biallelic HDR targeting using *piggyBac*-mediated selection removal (Jarazo et al.).

The ever-expanding repertoire of CRISPR editing systems includes the widely used Cas9 of *Streptococcus pyogenes* (*Sp*Cas9) (Jinek et al., 2012; Cong et al., 2013), as well as *Streptococcus thermophilus* (*St*Cas9) (Xu et al., 2015), and *Neisseria meningitides* (*Nm*Cas9) (Hou et al., 2013). In addition, other proteins in the CRISPR family such as Cpf1/Cas12a (Zetsche et al., 2015) have been applied for genome editing. More recently, CRISPR/Cas-derived novel genome editing tools that do not create DSBs have been developed, including the cytidine and adenine base editors (CBE and ABE) (Komor et al., 2016; Gaudelli et al., 2017), as well as prime editors (PE) (Anzalone et al., 2019). The paper by Wu et al. in this topic describes efforts to increase the CBE scope and efficiency in rice.

The rapid development of genome editing technology has provided opportunities for modifying large animal models and domestic animal breeding. Pigs serve as an important agricultural resource as well as animal models for biomedical studies. In this topic, Yang and Wu summarize the genome editing of pigs in agricultural and biomedical applications. Off-target effects are one of the major concerns for genome editing research. The last two articles (Li et al.; Zhou et al.) in this Research Topic report no obvious off-target events in the offspring of genetically edited goats. However, it remains to be determined as to whether these observations might be affected by survivorship bias, as well as differences in off-target events between human gene therapy and animal genetic breeding.

In conclusion, the articles contained within this Research Topic illustrate the mechanisms and great potential of precise genome editing techniques to further scientific inquiry and produce useful outcomes that benefit society.

## Author Contributions

KX wrote the manuscript draft. DS and ZZ supervised the topic and read the manuscript.

## Conflict of Interest

The authors declare that the research was conducted in the absence of any commercial or financial relationships that could be construed as a potential conflict of interest.
